# Inflammatory biomarkers in cardiac syndrome X: a systematic review and meta-analysis

**DOI:** 10.1186/s12872-024-03939-3

**Published:** 2024-05-28

**Authors:** Yuexia Zhao, Arshin Ghaedi, Pouria Azami, Seyed Ali Nabipoorashrafi, Hamed Bazrafshan Drissi, Maryam Amin Dezfouli, Shirin Sarejloo, Brandon Lucke-Wold, John Cerillo, Monireh Khanzadeh, Negar Jafari, Shokoufeh Khanzadeh

**Affiliations:** 1 Shandong Mental Health Center, Jinan, Shandong Province China; 2 Student Research Committee, School of Medicine, Shiraz University of Medical Sciences, Shiraz, Iran; 3 Cardiovascular Research Center, Shiraz University of Medical Sciences, Shiraz, Iran; 4 Endocrinology and Metabolism Research Center (EMRC), School of Medicine, Vali-Asr Hospital, Tehran, Iran; 5 Cardiovascular Department, Shiraz University of Medical Sciences, Shiraz, Iran; 6 Faculty of Medicine, Ahvaz Jundishapur University of Medical Sciences, Ahvaz, Iran; 7 Northern Health, Melbourne, VIC Australia; 8 Department of Neurosurgery, University of Florida, Gainesville, USA; 9 Nova Southeastern University Dr. Kiran C. Patel College of Osteopathic Medicine, Tampa Bay Regional Campus, Gulf to Bay Blvd, Clearwater, FL 3375 USA; 10 Geriatric & Gerontology Department, Medical School, Tehran University of medical and health sciences, Tehran, Iran; 11 Department of cardiovascular medicine, Tabriz University of Medical Sciences, Tabriz, Iran; 12 Tabriz University of Medical Sciences, Tabriz, Iran

**Keywords:** Cardiac syndrome X, Inflammation, Biomarker, Meta-analysis

## Abstract

**Introduction:**

In the current systematic review and meta-analysis, we aim to analyze the existing literature to evaluate the role of inflammatory biomarkers, including neutrophil-to-lymphocyte ratio (NLR), platelet-to-lymphocyte ratio (PLR), C-reactive protein (CRP), tumor necrosis factor-*a* (TNF-*a*), and interleukin-6 (IL-6) among individuals with cardiac syndrome X (CSX) compared to healthy controls.

**Methods:**

We used PubMed, Web of Science, Scopus, Science Direct, and Embase to systematically search relevant publications published before April 2, 2023. We performed the meta-analysis using Stata 11.2 software (Stata Corp, College Station, TX). So, we used standardized mean difference (SMD) with a 95% confidence interval (CI) to compare the biomarker level between patients and healthy controls. The I^2^ and Cochran’s Q tests were adopted to determine the heterogeneity of the included studies.

**Results:**

Overall, 29 articles with 3480 participants (1855 with CSX and 1625 healthy controls) were included in the analysis. There was a significantly higher level of NLR (SMD = 0.85, 95%CI = 0.55–1.15, I^2^ = 89.0 %), CRP (SMD = 0.69, 95%CI = 0.38 to 1.02, *p* < 0.0001), IL-6 (SMD = 5.70, 95%CI = 1.91 to 9.50, *p* = 0.003), TNF-*a* (SMD = 3.78, 95%CI = 0.63 to 6.92, *p* = 0.019), and PLR (SMD = 1.38, 95%CI = 0.50 to 2.28, *p* = 0.02) in the CSX group in comparison with healthy controls.

**Conclusion:**

The results of this study showed that CSX leads to a significant increase in inflammatory biomarkers, including NLR, CRP, IL-6, TNF-*a*, and PLR.

**Supplementary Information:**

The online version contains supplementary material available at 10.1186/s12872-024-03939-3.

## Introduction

Cardiac syndrome X (CSX) is characterized by typical or atypical anginal chest pain with no evidence of significant coronary vascular abnormalities visualized on the angiogram. The etiology of cardiovascular symptoms due to CSX has yet to be fully understood [1], and with previous studies failing to uncover specific pathophysiology of CSX, our abilities to cure and prevent this disease are limited. However, several pathogenic mechanisms have been proposed, including inflammation, neuroendocrine dysfunction, and oxidative stress [2, 3]. To further characterize immune dysregulation in CSX, many studies have evaluated levels of circulating inflammatory mediators, such as neutrophil to lymphocyte ratio (NLR), platelet to lymphocyte ratio (PLR), C-reactive protein (CRP), tumor necrosis factor-*a* (TNF-*a*), and interleukin-6 (IL-6) [4–32].

An emerging and unique inflammatory marker, the NLR, has more recently been investigated in the setting of human cardiac diseases like cancer, stroke, and hypertension. A population-based study on 13,732 participants published in 2024 showed that NLR was associated with coronary heart disease risk in adults [33]. Pathologically, blood neutrophils increase, and lymphocytes decrease in response to inflammatory stress, increasing the NLR. PLR, similar to NLR, is a relatively novel inflammatory marker, with recent studies showing significant elevation in several cardiac disorders [34]. For example, a recent meta-analysis published in 2024 showed that among heart failure patients, PLR was significantly lower in survived patients rather than deceased group [35]. In addition, CRP is an acute-phase protein released during times of increased stress and inflammation. Recent studies have shown that this biomarker can have some diagnostic and prognostic role in several cardiovascular disorders. For example, a 15-year prospective cohort study published in 2024 showed that CRP is associated with an increased risk of cardiovascular disease [36]. In addition, TNF-a and IL-6 are well-studied inflammatory markers released mostly by macrophages and monocytes during stressful events and have some diagnostic roles in cardiac disorders like hypertension and myocardial infarction [37–40]. While some previous studies have shown that these biomarkers were increased in CSX patients, other studies reported no association between these biomarkers and CSX; so, debate continues about the role of these biomarkers in CSX.

To better determine the association of NLR, CRP, IL-6, TNF-*a*, and PLR with CSX, we have conducted a meta-analysis to review the published literature examining inflammatory marker levels in these patient populations.

## Materials and methods

This study follows the Preferred Reporting Items for Systematic Reviews and Meta-analyses (PRISMA) 2020 reporting guideline [41]. The PRISMA checklist of this study is shown in Supplementary File A. The protocol was registered in PROSPERO (CRD42023448843).

### Eligibility criteria

Our inclusion criteria based on PICO criteria were as follows:

- Population: Patients with CSX.
- Control: Healthy controls.
- Intervention/Exposure: Level of NLR, CRP, IL-6, TNF-*a*, and PLR in CSX.
- Outcomes: Diagnostic significance of NLR, CRP, IL-6, TNF-*a*, and PLR in CSX.
- Study design: cohort, case-control, and cross-sectional studies.

Our exclusion criteria were as follows: (1) Those that did not compare any one of our outcomes inflammatory biomarkers (NLR, CRP, IL-6, TNF-a, OR PLR) levels between CSX patients and controls; (2) Studies that did not report their results as Mean ± standardized division (SD). There were not any limitations on language or date of publication.

### Information sources

One author (ShS) searched PubMed, Web of Science, Scopus, ScienceDirect, and Embase databases to identify all studies comparing the level of inflammatory biomarkers between CSX patients and healthy controls, published before April 2, 2023. Initially, we conducted a thorough search to identify all studies on the role of inflammatory biomarkers in CSX.

### Search strategy

Our search strategy was as follows: (“NLR“[All Fields] OR “neutrophil to lymphocyte ratio“[All Fields] OR “platelet to lymphocyte ratio“[All Fields] OR “PLR“[All Fields] OR “C-reactive protein“[All Fields] OR “CRP“[All Fields] OR “tumor necrosis factor“[All Fields] OR “TNF“[All Fields] OR “interleukin“[All Fields]) AND (“cardiac syndrome X“[All Fields] OR “microvascular angina“[MeSH Terms] OR “microvascular angina“[All Fields]). There was no limitation on the publication date or language of studies in our search. The exact search strategy is shown in Supplementary file B. We did not search unpublished studies.

Additionally, two authors (ShKh and ShS) reviewed the reference lists of included and relevant studies, according to the snowball method, to identify further eligible studies.

### Selection process

EndNote was used for study screening [42]. Initially, duplicate studies were deleted (521). Then, two authors (PA and ShS) screened the titles and abstracts of studies found in the initial search of databases and found the closely relevant studies (*N* = 85). Then, the same authors obtained and screened the full texts of these studies. Then, 41 studies were deleted due to lack of data on NLR, CRP, IL-6, TNF-a, or PLR, 11 studies due to irrelevant population, four for being review articles, and one due to lack of peer review. The remaining studies were used in the meta-analysis (*N* = 29). The kappa statistic was used to calculate Inter-reviewer agreement for the study selection [43]. The Kappa value of > 0.6 was considered a significant agreement between the authors.

### Data collection process

Supplementary file C shows our data extraction form. Two authors (MAD and PA) extracted data manually and independently using an Excel sheet.

### Data items

The first author, year of publication, study design, study location, total sample size, number of patients and healthy controls, type of biomarker measured, mean and SD of biomarker level, or any data for estimating the mean and SD (median and IQR or/and range) were extracted.

### Study risk of bias assessment

Two authors (MKh and MAD) conducted the quality assessment of included studies, utilizing the Newcastle–Ottawa scale (NOS) [44]. Disagreements between the authors were finally resolved via consensus and consulting with the third author(HB). Those studies with six or more points were deemed to have good quality.

### Effect measures

we used standardized mean difference (SMD) with a 95% confidence interval (CI) to compare the biomarker level between patients and healthy controls. The median(Interquartile range) values were converted to mean ± SD using the method introduced by Wan et al. [45].

### Synthesis methods

We performed the meta-analysis using Stata 11.2 software (Stata Corp, College Station, TX). The I^2^ and Cochran’s Q tests were adopted to determine the heterogeneity of the included studies. Significant heterogeneity between studies was conceived as I ^2^ >50% and *p*-value of the Q test < 0.05. In the case of significant heterogeneity, we used the random-effects model. Otherwise, the fixed-effect model was chosen. *P* value < 0.05 was considered significant.

### Reporting bias assessment

We used the funnel plot and Egger’s test to determine the publication bias. The symmetric plot was seen in the lack of bias.

### Certainty assessment

One of the authors (ShKh) utilized the Grading of Recommendations Assessment, Development, and Evaluation (GRADE) method to evaluate the certainty of the evidence for the outcome investigated in the meta-analysis [46].

## Results

### Search results and included studies

**Fig. 1:**
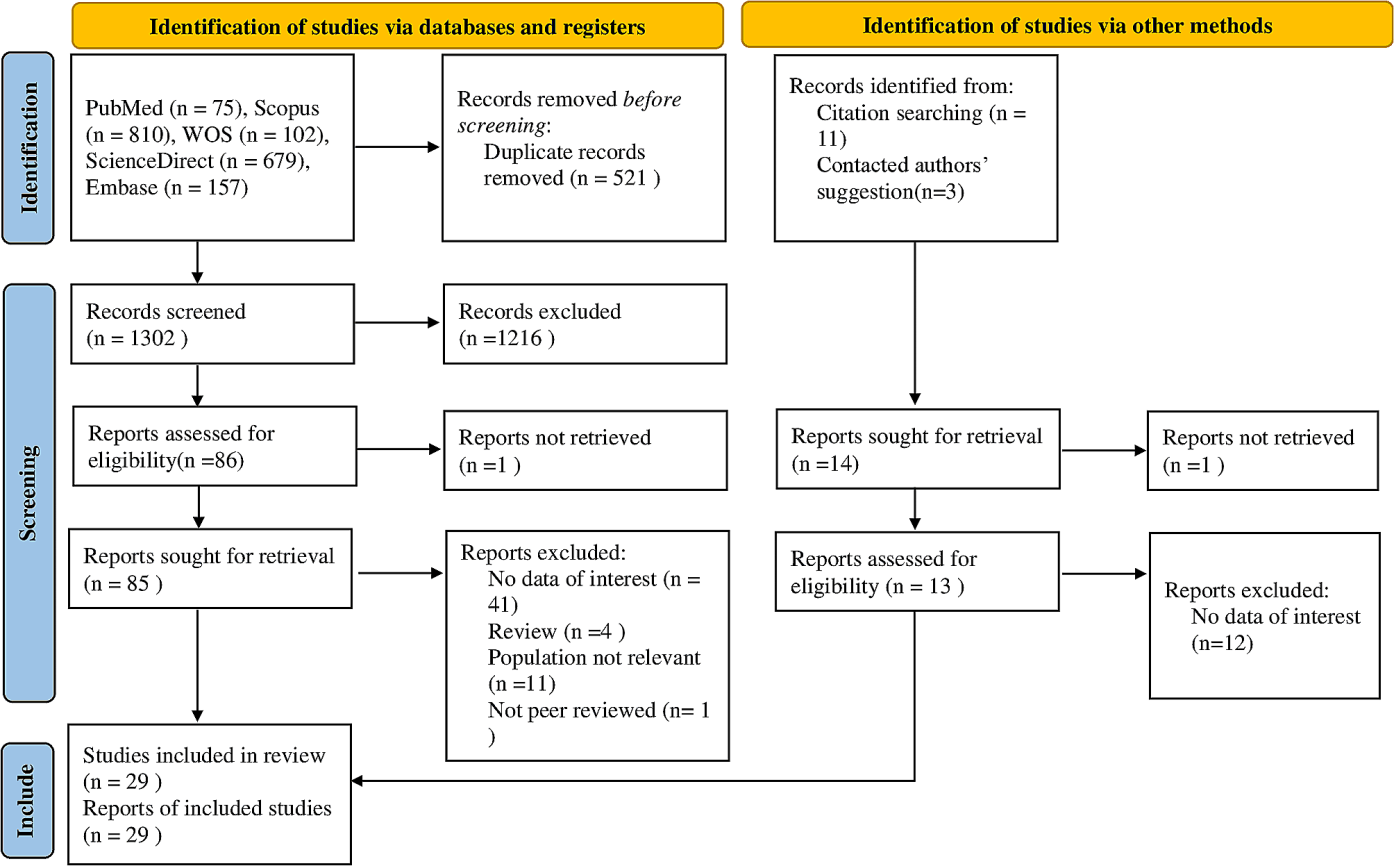
PRISMA 2020 Flow diagram for new systematic reviews which includes searches of databases, registers and other sources

Table 1 shows the general characteristics of included studies, and Fig. 1 shows the PRISMA flow diagram, indicating the process of inclusion and exclusion in detail [4–32]. We found an almost perfect agreement between the authors concerning interrater reliability of study selection (94% agreement; kappa = 0.83; 95%CI = 0.65–1.0, *P* < 0.001).

**Table 1:** General characteristics of included studies

First author	Year	Country	Design	CSX patients		Healthy controls		Adjusted variables in the included studies	NOS Score
				*N*	Biomarker level	*N*	Biomarker level		
Atmaca [7]	2008	Turkey	Prospective	59	NLR 2.75 ± 1.06	51	NLR 2.37 ± 0.65	Age, Sex, Hypertension, Diabetes Mellitus, Family history of CAD, Smoking, Medication	7
Demirkol [14]	2014	Turkey	Prospective	92	NLR 1.97 ± 0.58	99	NLR 1.78 ± 0.60	Alcohol consumption, BMI, Age, Sex, Hypertension, Diabetes Mellitus, Smoking, FBS	7
Yurtdas [31]	2014	Turkey	Prospective	135	NLR 3.10 ± 1.80	100	NLR 1.60 ± 1.10	Age, Sex, Hypertension, Dyslipidemia, EF	7
Okyay [23]	2015	Turkey	Retrospective	60	NLR 1.98 ± 0.77 PLR 133.0 ± 53.00	60	NLR 1.72 ± 0.55 PLR 120.00 ± 42.00	Age, Sex, Hypertension, Diabetes Mellitus, Smoking, Medication, Dyslipidemia, FBS, EF	6
Alizade [4]	2016	Turkey	Retrospective	50	NLR 2.80 ± 1.40	50	NLR 1.90 ± 1.20	Age, Sex, BMI, Hypertension, Diabetes Mellitus, Smoking, EF, FBS, Total plasma cholesterol, LDL, Medication	6
Altiparmak [5]	2016	Turkey	Prospective	50	NLR 2.20 ± 0.35	45	NLR 1.33 ± 0.23	Age, Sex, BMI, Hypertension, Diabetes Mellitus, Dyslipidemia, Smoking, Family history of CAD, Medication	8
Caglar [11]	2016	Turkey	Prospective	66	NLR 1.98 ± 0.66	47	NLR 1.67 ± 0.72	Sex, Diabetes Mellitus, Smoking, Family history of CAD, Dyslipidemia	6
Bolayir [8]	2017	Turkey	Retrospective	100	NLR 6.42 ± 0.78	100	NLR 5.74 ± 0.87	Age, Sex, BMI, Hypertension, Diabetes Mellitus, Dyslipidemia, Smoking	7
Boyraz [9]	2020	Turkey	Retrospective	80	NLR 2.67 ± 0.84 PLR 260.0 ± 16.00	80	NLR 1.87 ± 0.52 PLR 217.00 ± 14.00	Age, Sex, Hypertension, Diabetes Mellitus, Smoking, EF, Total plasma cholesterol, LDL	5
Cao [12]	2022	Turkey	Retrospective	102	NLR 1.92 ± 1.54 PLR 122.1 ± 30.78	102	NLR 1.41 ± 0.52 PLR 87.42 ± 21.16	Age, Sex, Hypertension, Diabetes Mellitus, Smoking	7
Yasar [30]	2022	Turkey	Retrospective	105	NLR 3.5 ± 1.1 130.0 ± 29.00	105	NLR 2.4 ± 0.9 102.00 ± 21.00	Age, Sex, Hypertension, Diabetes Mellitus, Dyslipidemia, Smoking, Medication, FBS, EF	6
Recio-Mayoral [26]	2013	Italy	Prospective	21	CRP 2.57 ± 2.26	21	CRP 0.73 ± 0.71	Age, Sex, BMI, Hypertension, Total plasma cholesterol, TG, FBS	7
Arroyo-Gspliguero [6]	2003	United Kingdom	Prospective	30	CRP 2.93 ± 2.17	30	CRP 1.63 ± 1.55	Age, Sex, BMI, Hypertension, TG Smoking, Medication, FBS, Family history of CAD	6
Eroglu [16]	2009	Turkey	Prospective	100	CRP 5.50 ± 1.10	50	CRP 4.40 ± 1.20	Age, Sex, Hypertension, Diabetes Mellitus, Dyslipidemia, Smoking, Medication, FBS, EF	7
Li [20]	2007	China	Prospective	36	CRP 0.48 ± 0.26 IL-6 3.40 ± 1.20	30	CRP 0.22 ± 0.15 IL-6 6.20 ± 0.60	Age, Sex, BMI, Hypertension, Diabetes Mellitus, Hyperlipidemia, Smoking, Family history of CAD, EF	6
Lanza [19]	2004	Italy	Prospective	43	CRP 4.06 ± 6.80	39	CRP 1.75 ± 1.98	Age, Sex, Diastolic BP, Smoking, Total plasma cholesterol, LDL	6
Tondi [28]	2011	Italy	Prospective	42	CRP 2.75 ± 2.40	20	CRP 0.74 ± 0.40	Age, Sex, Hypertension, Glucose intolerance, Hypercholesterolemia, Smoking, Family history of CAD	6
Karakas [18]	2012	Turkey	Prospective	35	CRP 1.03 ± 0.50	34	CRP 0.71 ± 0.49	Age, Sex, BMI, Hypertension, Smoking, EF, Medication	6
Lin [21]	2003	China	Prospective	32	TNF 56.90 ± 22.40	17	TNF 54.10 ± 22.60	Age, Sex, BMI, Hypertension, Smoking, HDL, LDL, Glucose	7
Rasmi [25]	2012	Iran	Prospective	60	IL-6 33.60 ± 3.50 TNF 24.20 ± 2.30	60	IL-6 3.20 ± 0.40 TNF 3.10 ± 0.40	Age, Sex, BMI, Hypertension	7
Demir [13]	2016	Turkey	Prospective	111	CRP 0.34 ± 0.61 IL-6 42.70 ± 61.30 TNF 83.10 ± 2.30	111	CRP 0.27 ± 0.54 IL-6 22.30 ± 24.50 TNF 84.90 ± 3.50	Age, Sex, BMI, Hypertension, Diabetes Mellitus, Dyslipidemia, Smoking, Medication, Glucose, Family history of CAD	7
Mahfouz [22]	2016	Egypt	Prospective	54	CRP 6.38 ± 2.25 IL-6 7.85 ± 1.90 TNF 6.18 ± 1.62	32	CRP 1.29 ± 0.07 IL-6 2.91 ± 0.85 TNF 1.75 ± 0.42	Age, BMI, Hypertension, Glucose	6
Guler [17]	2014	Turkey	Prospective	50	CRP 0.63 ± 0.39	33	CRP 0.50 ± 0.36	Age, BMI, waist circumference, Hypertension, Diabetes Mellitus, metabolic syndrome, hyperlipidemia, Smoking, FBS, EF	7
Ungan [29]	2019	Turkey	Prospective	51	CRP 2.10 ± 4.80	46	CRP 1.60 ± 3.70	Sex, BMI, Hypertension, Smoking, HDL, total cholesterol	6
Buyukkaya [10]	2013	Turkey	Prospective	41	CRP 1.04 ± 0.45	40	CRP 0.73 ± 0.51	Age, Sex, BMI, Hypertension, Diabetes Mellitus, EF, Smoking, Medication, Dyslipidemia, Glucose	6
Sahin [27]	2012	Turkey	Prospective	33	CRP 1.06 ± 0.30	20	CRP 0.38 ± 0.15	Age, Sex, BMI, Hypertension, Diabetes Mellitus, FBS, Smoking, Medication, Dyslipidemia	7
Qing [24]	2013	China	Prospective	120	CRP 2.80 ± 2.20	102	CRP 2.00 ± 1.70	Age, Sex, BMI, Hypertension, Diabetes Mellitus, EF, Smoking, Medication, Dyslipidemia	8
Akin [32]	2023	Turkey	Retrospective	80	NLR 1.83 ± 0.26 CRP 4.66 ± 3.01	80	NLR 1.55 ± 0.29 CRP 2.93 ± 2.71	Age, Sex, BMI, Hypertension, Diabetes Mellitus, Smoking, Dyslipidemia, Family history of CAD, Glucose, EF	7
Dollard [15]	2016	Ireland	Prospective	17	NLR 2.22 ± 0.09 CRP 2.28 ± 3.50	21	NLR 1.47 ± 0.08 CRP 0.69 ± 0.81	BMI, Age, Sex, Hypertension, Diabetes Mellitus, Smoking, Medication, Dyslipidemia	7

R: Retrospective; P: Prospective; NLR: Neutrophil to lymphocyte ratio; NOS: Newcastle-Ottawa scale, PLR: Platelet to lymphocyte ratio; CRP: C-Reactive protein; IL-6: Interleukin-6; TNF: Tumor necrosis factor ; LDL: Low density lipoprotein ; HDL: high density lipoprotein; BMI: body mass index; EF, ejection fraction; FBS: Fasting blood sugar

### NLR level in patients with cardiac syndrome X

**Fig. 2:**
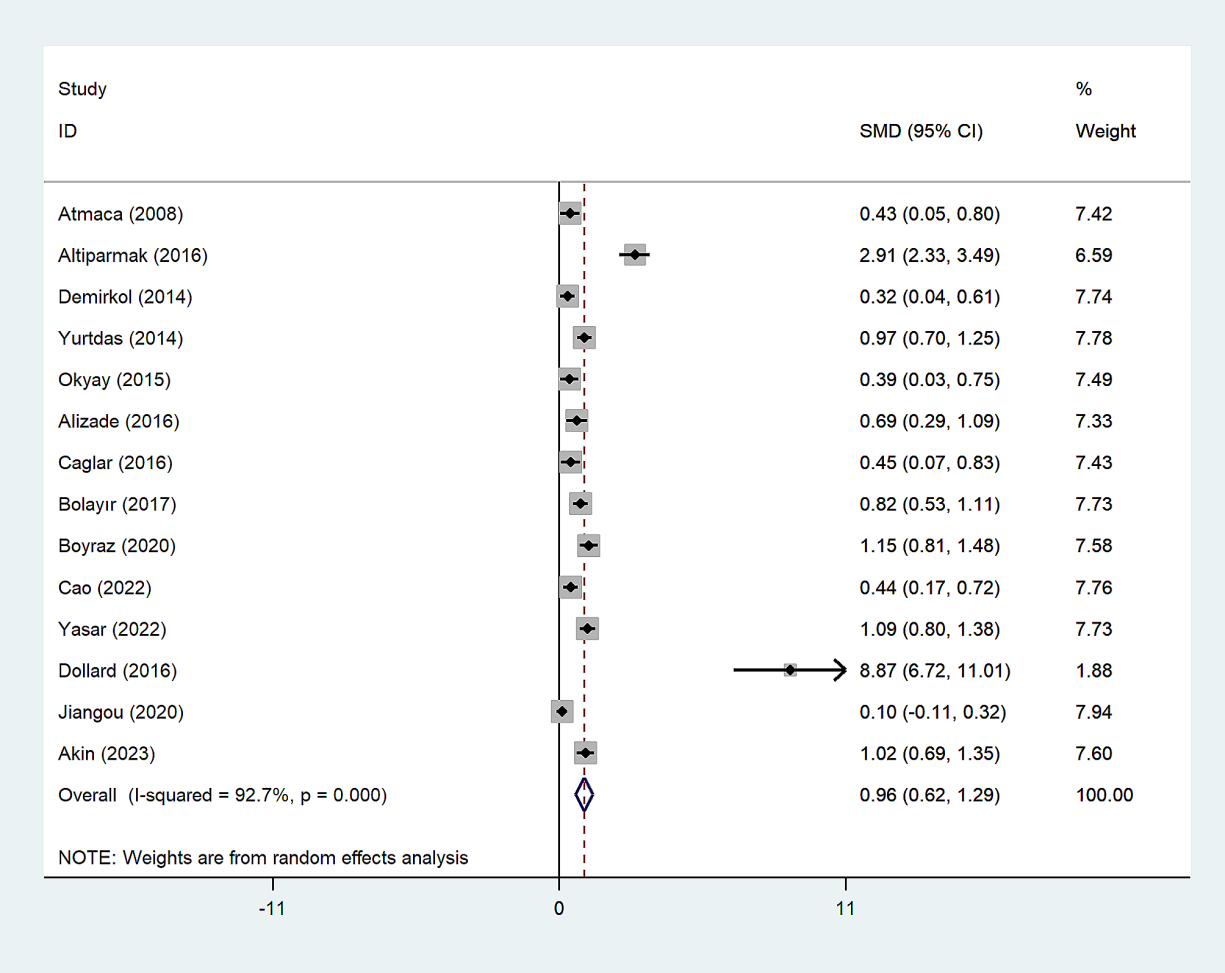
Meta-analysis of differences in NLR level between CSX patients and healthy controls

A random-effect model revealed significantly higher NLR levels in the CSX group than healthy controls (SMD = 0.85, 95%CI = 0.55 to 1.15, *p* < 0.0001, I^2^ = 89.0%) (Fig. 2). However, the GRADE approach determined that the certainty of this summary estimate of effect was very low. (Table 2).

**Table 2:** Grade^1^ evidence profile for studies on the role of neutrophil to lymphocyte ratio in cardiac syndrome X

Certainty assessment							No of patients		Certainty^6^	Importance
No of studies	Study design	Risk of bias^2^	Inconsistency^3^	Indirectness	Imprecision^4^	Publication bias^5^	Participants, *n*	Cases, *n*		
NLR										
13	observational studies	not serious	very serious	not serious	not serious	very serious	1936	996	⨁◯◯◯ Very low	CRITICAL
CRP										
21	observational studies	not serious	very serious	not serious	not serious	very serious	2266	1224	⨁◯◯◯ Very low	CRITICAL
IL-6										
4	observational studies	not serious	very serious	not serious	not serious	very serious	494	261	⨁◯◯◯ Very low	CRITICAL
TNF-a										
4	observational studies	not serious	very serious	not serious	not serious	not serious	477	257	⨁◯◯◯ Very low	CRITICAL
PLR										
4	observational studies	not serious	very serious	not serious	not serious	not serious	694	347	⨁◯◯◯ Very low	CRITICAL

^1^Grading of Recommendations Assessment, Development and Evaluation

^2^Risk of bias based on Newcastle-Ottawa Scale

^3^When I^2^ was < 30% inconsistency considered as Not serious limitation, > 50 considered as serious and more than 75% considered as very serious limitation

^4^Serious limitations when there was fewer than 400 participants for each outcome and very serious limitations when there was fewer than 300 participants for each outcome

^5^Funnel plot revealed some evidence of asymmetry and test of publication bias approached *P* < 0.10

^6^Data from cohort studies begin with a grade of “LOW”. Downgraded for very serious inconsistency

**Fig. 3:**
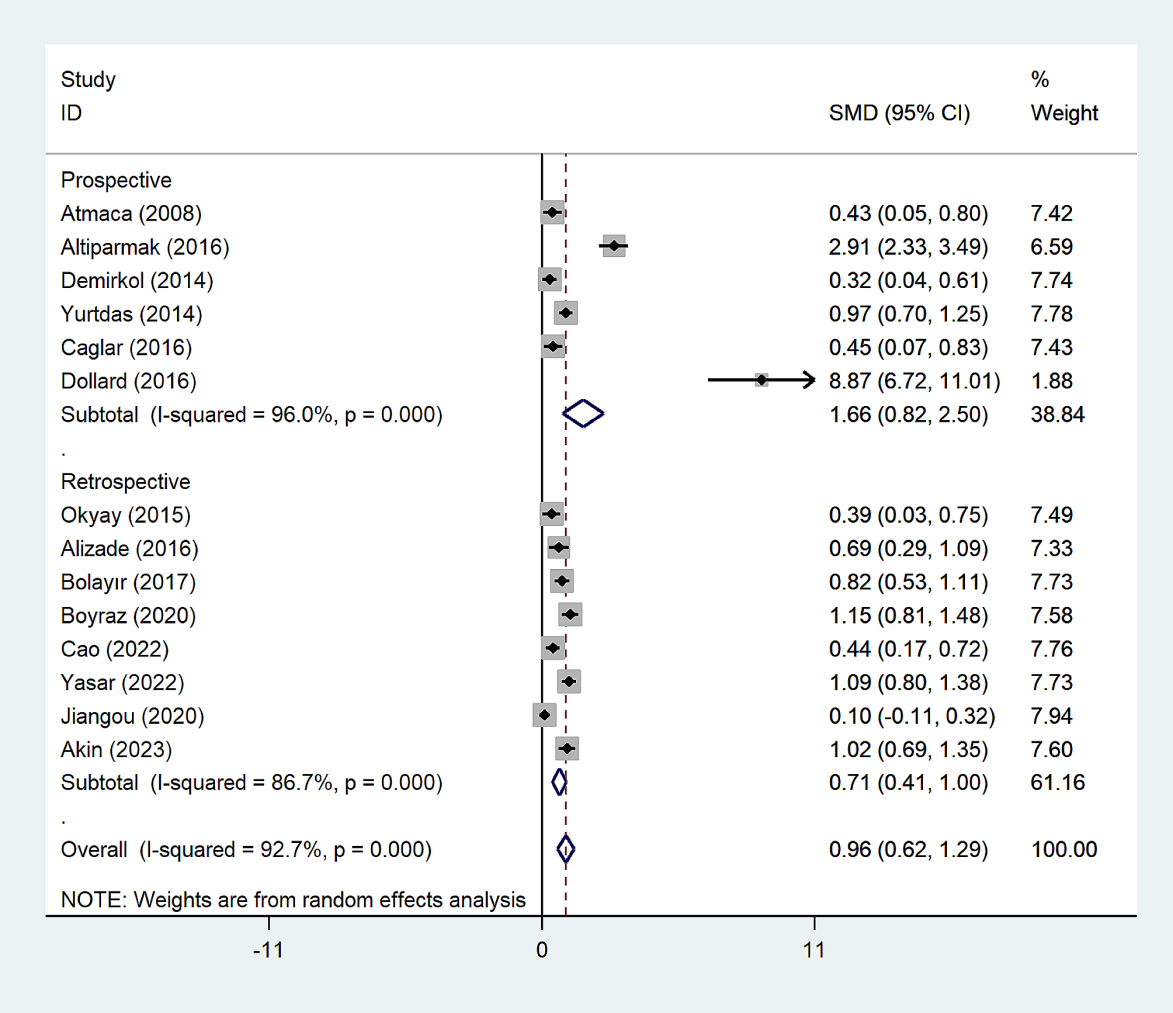
Subgroup analysis of differences in NLR level between CSX patients and healthy controls according to study design

The subgroup analysis according to the study design demonstrated that there was a significantly higher NLR level among CSX patients in comparison with healthy controls in either retrospective (SMD = 0.83, 95% CI = 0.51 to 1.14, *p* < 0.001, I^2^ = 74.3%) or prospective studies (SMD = 0.98, 95% CI = 0.32 to 1.64, *p* = 0.004, I^2^ = 94.2%) (Fig. 3).

### CRP level in patients with cardiac syndrome X

**Fig. 4:**
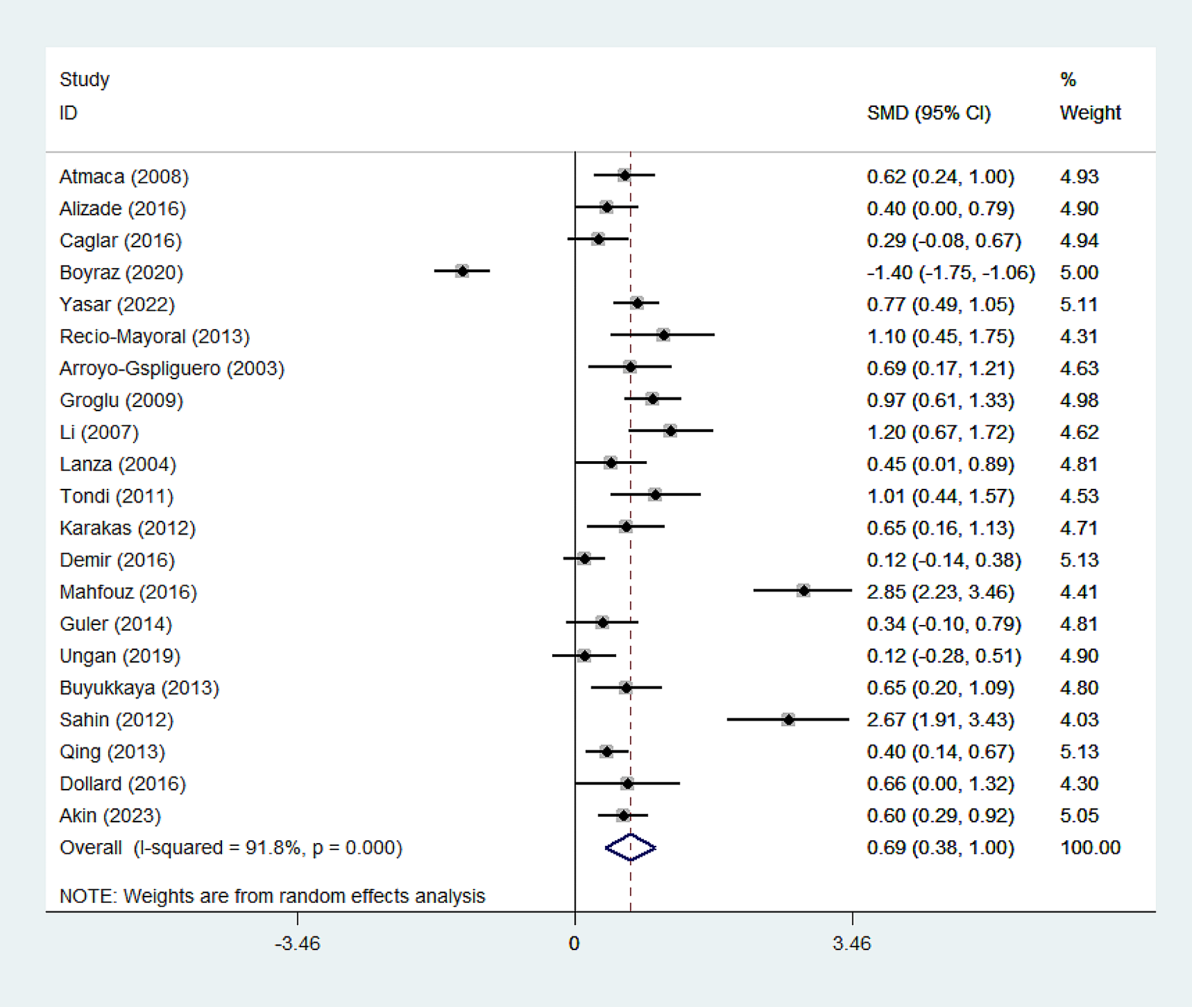
Meta-analysis of differences in CRP level between CSX patients and healthy controls

CRP level was significantly higher in the CSX group than healthy controls (SMD = 0.69, 95%CI = 0.38 to 1.02, *p* < 0.0001). A random-effect model was used due to high heterogeneity (I^2^ = 92.2%, Fig. 4). However, the GRADE approach determined that the certainty of this summary estimate of effect was very low (Table 2).

### IL-6 level in patients with cardiac syndrome X

**Fig. 5:**
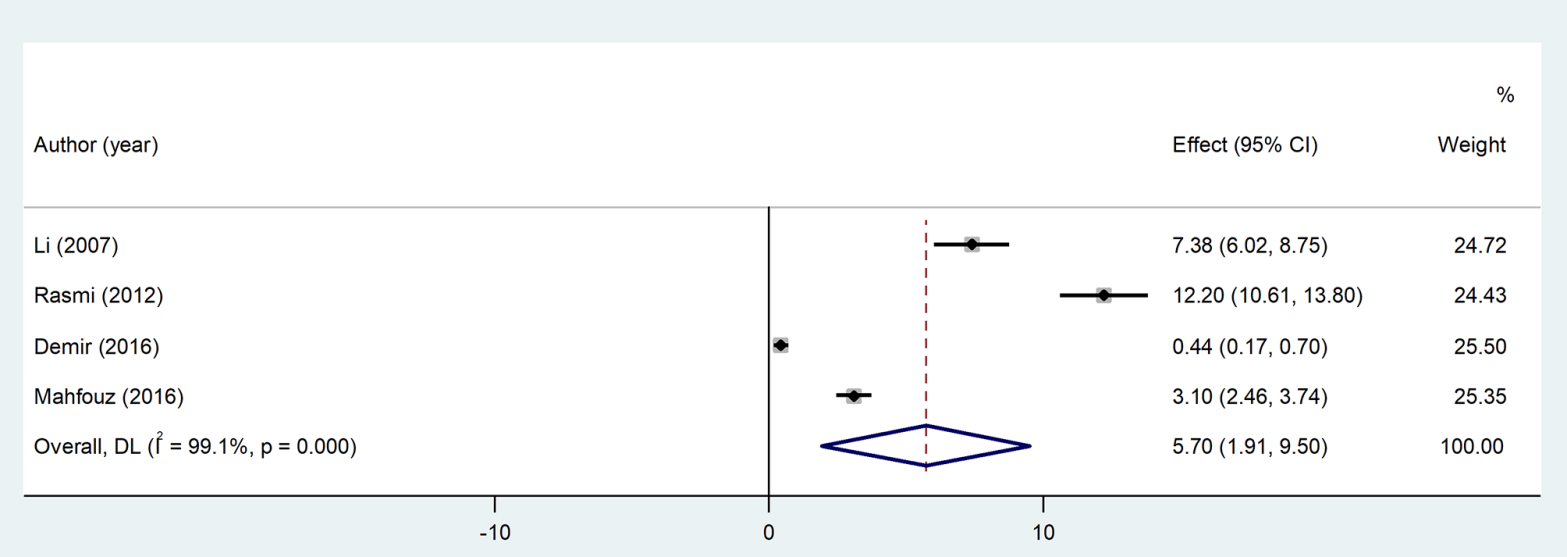
Meta-analysis of differences in IL-6 level between CSX patients and healthy controls

Meta-analysis of four relevant articles using the random-effect model showed that IL-6 level was significantly higher in the CSX group compared to healthy controls (SMD = 5.70, 95%CI = 1.91 to 9.50, *p* = 0.003, I^2^ = 99.1%, Fig. 5). According to the GRADE approach, the certainty of this summary estimate of effect was very low (Table 2).

### TNF-*a* level in patients with Cardiac Syndrome X

**Fig. 6:**
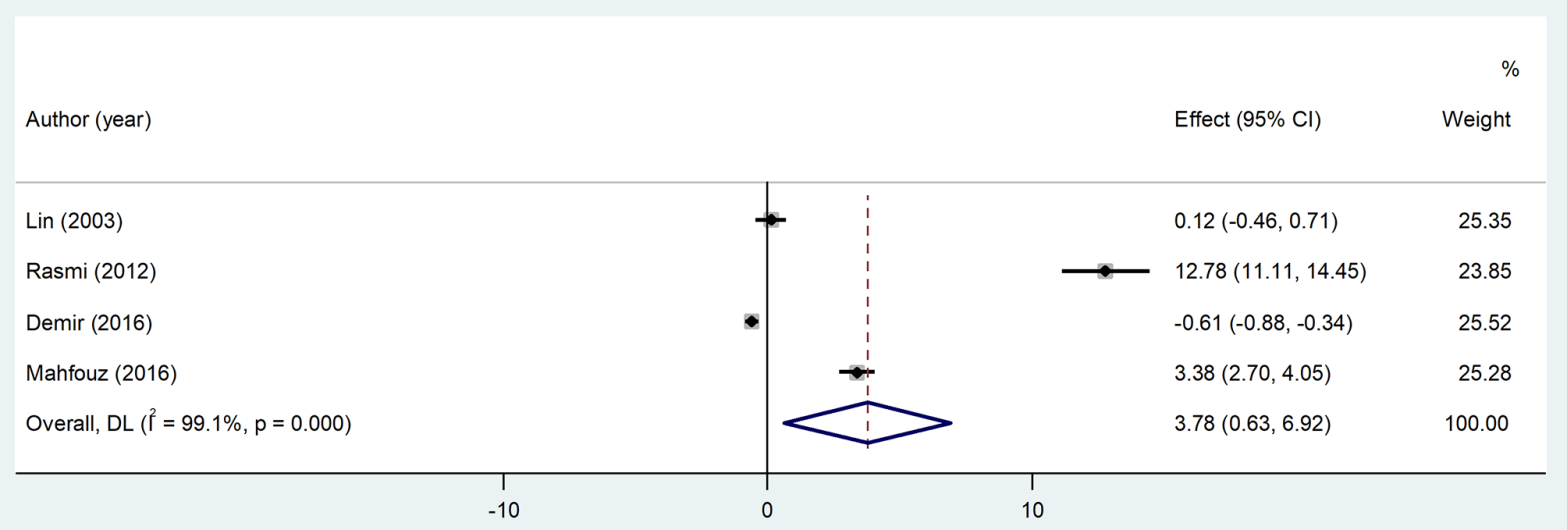
Meta-analysis of differences in TNF-*a* level between CSX patients and healthy controls

In this meta-analysis of four relevant articles, TNF-*a* level was significantly higher in the CSX group (random-effect model, SMD = 3.78, 95%CI = 0.63 to 6.92, *p* = 0.019, I^2^ = 99.1%, Fig. 6). The certainty of this summary effect estimate was very low (Table 2).

### PLR Level in patients with cardiac syndrome X

**Fig. 7:**
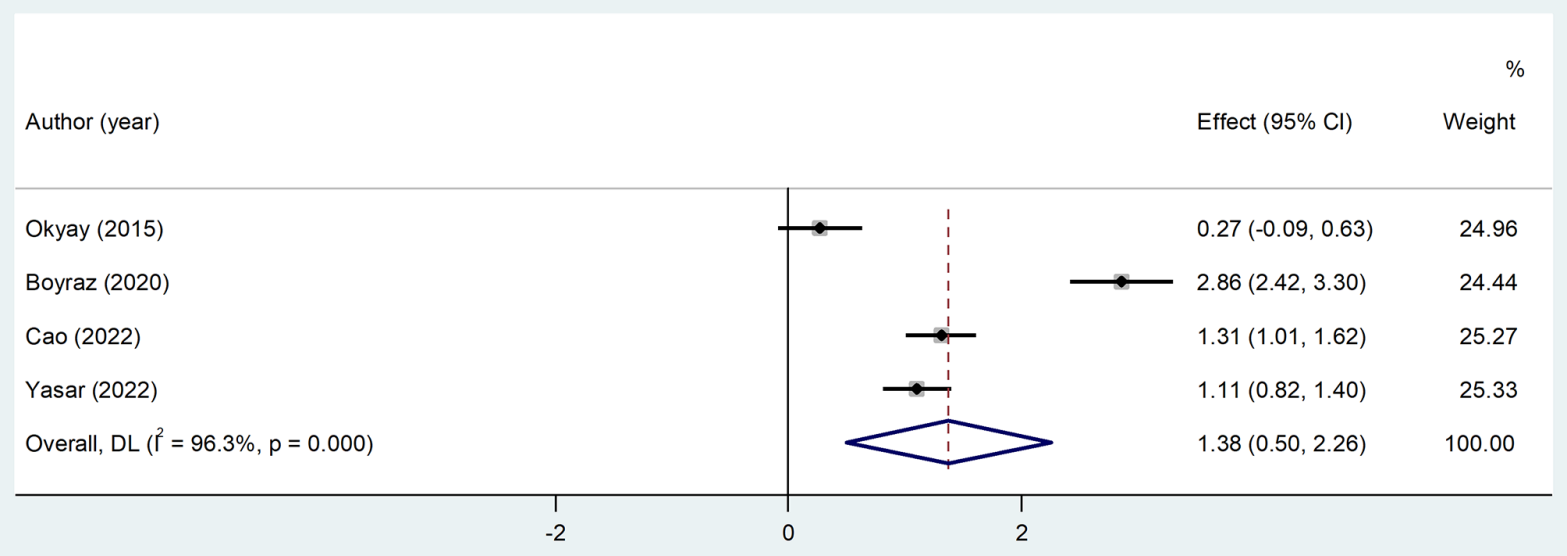
Meta-analysis of differences in PLR level between CSX patients and healthy controls

Then we analyzed the differences in PLR level between CSX patients and healthy controls and found that compared to healthy controls, PLR level was significantly higher in the CSX group (random-effect model, SMD = 1.38, 95%CI = 0.50 to 2.28, *p* = 0.02, I^2^ = 96.3%, Fig. 7). However, the certainty of evidence was very low in this analysis (Table 2).

### Publication bias

Egger’s test showed no publication bias among studies on PLR(*p* = 0.47) and TNF-a (*p* = 0.07). However, there was some evidence of potential publication bias among studies on NLR(*p* = 0.01), CRP (*p* = 0.02), and IL6 (*p* = 0.01). The relevant funnel plots are shown in supplementary File D.

## Discussion

CSX is a significant cause of morbidity due to recurring angina events despite its favorable long-term prognosis. This condition’s pathophysiology must be understood to offer patients the best possible treatment. Although the pathophysiology of CSX is complex, inflammation is likely a key factor [2]. This meta-analysis showed a significant increase in NLR level as a new inflammatory biomarker in CSX patients compared to healthy controls. This difference remained significant in a subgroup analysis by study design. The analysis also showed significant increases in CRP, IL-6, TNF-*a*, and PLR levels reported in prior studies.

Neutrophils play a central role in innate immunity by enhancing pro-inflammatory reactions, while lymphocytes are integral to the adaptive immune system, modulating immune responses. When NLR is elevated, the inflammatory actions of neutrophils may surpass the regulatory effects of lymphocytes, leading to a notable escalation in peripheral inflammation [47].

Numerous original studies have been published on the role of NLR and inflammatory markers in recent literature, and there is now a need for a systematic review of the literature. For example, a recent meta-analysis indicated that an elevated pretreatment NLR could predict the risk of major adverse cardiac events (MACE) and mortality in patients who had a recent acute coronary syndrome [48]. Another meta-analysis reported that NLR could predict arrhythmia, in-stent thrombosis, angina, no-reflow, advanced heart failure, nonfatal myocardial infarction, long-term all mortality, cardiac mortality, MACE, and nonfatal myocardial infarction (MI) in patients with acute ST-segment elevation MI after percutaneous coronary intervention [49]. In addition, a meta-analysis conducted by Liu et al. has shown that elevated preoperative NLR could predict postoperative atrial fibrillation [50]. In another meta-analysis, an elevated preoperative NLR (> 5 in vascular surgery, > 3.3 in cardiac surgery) was associated with high mortality at a mean follow-up of 34.8 months, raised risk of post-operative re-intubation, amputation in vascular operations, and increased cardiac mortality [51]. In the recent meta-analysis on the association of NLR with heart failure, NLR was associated with all-cause mortality and renal dysfunction [52].

To the best of our knowledge, there has not been a review of research on CSX. Our findings are the first to provide a thorough and up-to-date assessment of NLR and other inflammatory marker levels in CSX patients based on a review of all publications.

Despite numerous studies investigating the correlation between NLR and CSX in the past, the findings have been inconsistent. The existing literature has shown a significant variability in the reported associations between NLR and CSX. While dissecting the results, we found that NLR has been shown in the literature to be a unique inflammatory marker that may reflect important immunologic abnormalities critical to the development of CSX [31].

The PLR is increasingly being viewed as a marker of systemic inflammation due to studies indicating that platelets have early involvement in the inflammatory response and tissue healing [53]. However, studies on the PLR are less frequent compared to studies on the NLR [54]. Platelets collaborate with different types of white blood cells and release substances that prompt leukocytes to adhere faster to endothelial surfaces, potentially causing cellular leakage. Platelets can significantly impact the inflammatory response of leukocytes, either enhancing or suppressing their activity. An increased PLR may result from chronic low-grade inflammation, and a rising PLR could indicate persistent inflammation, increasing susceptibility to various conditions like CSX, coronary artery disease, autoimmune diseases, and solid organ tumors [55].

Similar to the NLR, many studies have investigated the potential of the PLR as a diagnostic tool for determining the extent of inflammation in various cardiovascular diseases. Recently, a meta-analysis revealed that an increased PLR may indicate stable CAD and can help predict collateral circulation, severe CAD stenosis, and CSF [56]. Higher PLR predicts worse in-hospital and long-term outcomes in STEMI patients following pPCI, according to a meta-analysis published in 2021 [57]. In another study, Wang and colleagues discovered that NLR and PLR are potential biomarkers for predicting prognosis in individuals with acute pulmonary embolism (PE) since the increase of both biomarkers was linked to greater mortality rates [58]. A meta-analysis conducted in 2021 found that patients with STEMI who have higher PLR levels upon admission and receive primary PCI treatment are more likely to experience in-hospital MACE and mortality, as well as long-term MACE and mortality [59]. Lastly, Li and colleagues discovered that PLR has the potential to be an effective biomarker to predict the likelihood of poorer prognosis in patients with ACS [60]. Our study also showed significantly higher levels of PLR in CSX patients.

The positive results from different studies indicate that NLR and PLR are proper biomarkers for cardiovascular diseases, and their elevation can predict adverse outcomes. However, the specificity of NLR and PLR for these diseases is under debate as they may not differentiate two different illnesses. Aging, ethnicity, obesity, and genetic factors are other factors that can alter inflammatory biomarkers, so clinicians should be cautious when using these biomarkers for their diagnosis [61, 62].

Other investigations have shown an unequal distribution of pro-inflammatory elements and anti-inflammatory processes in individuals with CSX, suggesting that inflammation could contribute to endothelial and microvascular dysfunction, which are believed to play a role in the onset of CSX [2]. Tousoulis and colleagues found elevated levels of intercellular adhesion molecule-1 and vascular cell adhesion molecule-1 (VCAM-1) in the bloodstream of patients with CSX. These molecules are produced by activated endothelial cells in response to inflammatory triggers [63].

As for IL6 and TNF-*a*, similar elevation was found in our analysis of CSX patient studies.

IL-6 is a significant cytokine that has both anti-inflammatory and pro-inflammatory effects. It can be produced locally by crucial cells in the development of atherosclerosis, such as muscle cells, endothelial cells, and macrophages [26]. Therefore, it can lead to vascular inflammation by inducing the formation of foam cells in macrophages, dysfunction of the endothelium, migration of inflammatory cells into the subintima, and smooth muscle proliferation [64]. This indicates that IL-6 may contribute to the pathogenesis of CSX by promoting inflammation and initiating endothelial dysfunction, which leads to reduced vascular reactivity, specifically microvascular vasoconstriction. It may also cause myocardial ischemia in CSX by increasing diffuse atherosclerosis at the microvasculature [65].

TNF-a has been associated with the development and advancement of atherosclerosis and its related outcomes, such as acute coronary syndrome. Additionally, TNF-a has been demonstrated to hinder the generation of NO by inhibiting the production of the endothelial nitric oxide synthase enzyme in endothelial cells [66]. The most commonly accepted explanation of CSX pathogenesis suggests that this condition can induce endothelial dysfunction, leading to impaired microvascular function.

Notably, new evidence suggests that CRP may be both a marker and a mediator of cardiac syndrome X. CRP induces endothelial cells to produce cellular adhesion molecules, endothelin-1, and interleukin-6 [25]. CRP plays a role in activating monocyte chemoattractant protein-1 and enhancing the uptake of low-density lipoprotein by macrophages. Additionally, CRP inhibits angiogenesis, decreases nitric oxide synthesis, upregulates the angiotensin type 1 receptor in smooth muscle cells, and reduces prostacyclin release by endothelial cells [67]. Reducing CRP levels by using statins or aspirin could improve coronary microvascular function. However, the efficacy and safety of this approach are not definitively established and require further investigation. Indeed, since many inflammatory mediators exist, identifying inflammatory mechanisms and triggers in each specific clinical scenario and targeting therapy to the particular rate limiting or trigger stages in effector pathways looks more plausible [67].

While patients with CSX have a favorable prognosis, they frequently develop recurring angina pectoris crises. Several drugs used to treat stenotic coronary artery disease can be recommended in this clinical state; however, there is no specific treatment available due to the unknown etiology of the disease [1]. We believe future research into specific CSX treatment approaches will concentrate on novel pathways, such as targeting inflammation and NLR levels.

### Limitations and strengths

We gathered all information on the association between inflammatory biomarkers and cardiac syndrome X for the current meta-analysis. Although meta-analysis often strengthens the existing evidence, there are various limitations to consider when interpreting our study results. Our study has several limitations associated with the observational nature of the included studies with all the inherited biases. In fact, the considerable heterogeneity found, which is likely due to the inclusion criteria used by the participants, as well as the study designs, diagnostic criteria, sex, and age of participants, may have led to indefinite findings. Furthermore, the results could have been biased due to publication bias. Furthermore, using the GRADE method, the GRADE approach determined that the certainty of this summary estimate of effect was very low. Another limitation was that most of the included studies were conducted in Turkey, China, and Italy. Inflammatory biomarkers may differ depending on race and other factors. As a result, further studies in other regions are needed to verify or reject the influence of race on inflammatory biomarkers. In addition, our findings are limited by the use of SMD instead of odds ratio(OR), risk ratio (RR), and hazard ratio (HR). When we extracted the data, most relevant studies reported mean ± SD. So, in our study, SMD was used. This limitation means that study findings must be interpreted cautiously because average values can prove differences but not correlations. This issue is intriguing and could be usefully explored in further research. Despite these limitations, our findings provide crucial clinical implications. To the best of our knowledge, this is the first meta-analysis that completely analyzes information addressing the relationship between inflammatory biomarkers and cardiac syndrome X. Other key strengths of our meta-analysis should also be highlighted. First, we established a repeatable and thorough search method for each database, in addition to the manual reference search of the references of the first selected papers, reviews, meta-analyses, or comments. Furthermore, various inflammatory biomarkers were carefully analyzed in this study, although further research is required to determine a cut-off value point for such biomarkers. Finally, all the included articles excluded the patients with disorders affecting inflammatory markers, such as hematological disorders, chronic or acute inflammatory or infectious diseases, malignancies, hepatic insufficiency, renal dysfunction, or steroid therapy. Regarding the fact that in several systemic disorders, the level of inflammatory markers may rise, and it can affect the results of diagnostic tests, this exclusion criterion among included studies could substantially increase our results’ validity.

## Conclusion

Despite the limitations, our results showed that the levels of NLR, CRP, IL-6, TNF-*a*, and PLR in patients with CSX were increased compared to healthy controls. Although these biomarkers are straightforward and readily accessible, making them potentially suitable options for countries with limited healthcare resources, their ability to predict CSX needs further exploration. Additional studies are necessary to investigate the correlation between these ratios and CSX in more depth.

### Electronic supplementary material

Below is the link to the electronic supplementary material.

Supplementary Material 1: supplementary File D shows the relevant funnel plots.

Supplementary Material 2: Supplementary file B shows the exact search strategy of databases.

Supplementary Material 3: Supplementary file A shows PRISMA checklist.

Supplementary Material 4: Supplementary file C shows our data extraction form

## Data Availability

The dataset supporting the conclusions of this article is included in the article.

## Abbreviations

neutrophil to lymphocyte ratio: NLR
platelet to lymphocyte ratio: PLR
C-reactive protein: CRP
tumor necrosis factor-*a*: TNF-*a*
interleukin-6: IL-6
cardiac syndrome X: CSX
vascular cell adhesion molecule-1: VCAM-1
Standardized mean difference: SMD
95% confidence interval: 95% CI
