# Correction: Inflammatory biomarkers in cardiac syndrome X: a systematic review and meta-analysis

**DOI:** 10.1186/s12872-024-03968-y

**Published:** 2024-06-08

**Authors:** Yuexia Zhao, Arshin Ghaedi, Pouria Azami, Seyed Ali Nabipoorashrafi, Hamed Bazrafshan Drissi, Maryam Amin Dezfouli, Shirin Sarejloo, Brandon Lucke-Wold, John Cerillo, Monireh Khanzadeh, Negar Jafari, Shokoufeh Khanzadeh

**Affiliations:** 1 Shandong Mental Health Center, Jinan, Shandong Province China; 2 Student Research Committee, School of Medicine, Shiraz University of Medical Sciences, Shiraz, Iran; 3 Cardiovascular Research Center, Shiraz University of Medical Sciences, Shiraz, Iran; 4 Endocrinology and Metabolism Research Center (EMRC), School of Medicine, Vali-Asr Hospital, Tehran, Iran; 5 Cardiovascular Department, Shiraz University of Medical Sciences, Shiraz, Iran; 6 Faculty of Medicine, Ahvaz Jundishapur University of Medical Sciences, Shirin Sarejloo, Ahvaz, Iran; 7 Northern Health, Melbourne, VIC Australia; 8 Department of Neurosurgery, University of Florida, Gainesville, USA; 9 Nova Southeastern University Dr. Kiran C. Patel College of Osteopathic Medicine, Tampa Bay Regional Campus, Gulf to Bay Blvd, Clearwater, FL 3375 USA; 10 Geriatric & Gerontology Department, Medical School, Tehran University of medical and health sciences, Negar Jafari, Tehran, Iran; 11 Department of cardiovascular medicine, Tabriz University of Medical Sciences, Shokoufeh Khanzadeh, Tabriz, Iran; 12 Tabriz University of Medical Sciences, Tabriz, Iran

**BMC Cardiovascular Disorders (2024) 24:276**

10.1186/s12872-024-03939-3

Following publication of the original article [1], the affiliation details for Yuexia Zhao was incorrectly given as ‘Shandong Medical Health Center, Jinan, Shandong Province, China’ but should have been ‘Shandong Mental Health Center, Jinan, Shandong Province, China’.

The original article has been corrected.
