# Supplementary material for: Inflammatory biomarkers in cardiac syndrome X: a systematic review and meta-analysis
Source: BMC Cardiovasc Disord. 2024 May 28;24:276. doi: 10.1186/s12872-024-03939-3 (PMC11134643; doi:10.1186/s12872-024-03939-3)
Supplement: Supplementary file 1 — Supplementary Material 1: supplementary File D shows the relevant funnel plots. [file 12872_2024_3939_MOESM1_ESM.docx]

**Supplemental Figure I.** Funnel plot assessing publication bias in articles about NLR in CSX patients

**Supplemental Figure II.** Funnel plot assessing publication bias in articles about TNF-α in CSX patients

**Supplemental Figure III.** Funnel plot assessing publication bias in articles about PLR in CSX patients

**Supplemental Figure IV.** Funnel plot assessing publication bias in articles about CRP in CSX patients

**Supplemental Figure V.** Funnel plot assessing publication bias in articles about IL-6 in CSX patients
