# Supplementary material for: Inflammatory biomarkers in cardiac syndrome X: a systematic review and meta-analysis
Source: BMC Cardiovasc Disord. 2024 May 28;24:276. doi: 10.1186/s12872-024-03939-3 (PMC11134643; doi:10.1186/s12872-024-03939-3)
Supplement: Supplementary file 2 — Supplementary Material 2: Supplementary file B shows the exact search strategy of databases. [file 12872_2024_3939_MOESM2_ESM.docx]

**Supplementary file A:** The exact search strategy of databases.

Scopus

N=810

( TITLE-ABS-KEY ( nlr OR ( neutrophil AND to AND lymphocyte AND ratio ) OR ( platelet AND to AND lymphocyte AND ratio ) OR plr OR ( c-reactive AND protein ) OR crp OR ( tumor AND necrosis AND factor ) OR tnf OR interleukin ) AND TITLE-ABS-KEY ( ( cardiac AND syndrome AND x ) ) )

WOS

N=102

NLR OR "neutrophil to lymphocyte ratio" OR "platelet to lymphocyte ratio" OR PLR OR "C-reactive protein" OR CRP OR "tumor necrosis factor" OR TNF OR interleukin (All Fields) and "Cardiac syndrome X" (All Fields)

SienceDirect

N=679

NLR OR "neutrophil to lymphocyte ratio" OR "platelet to lymphocyte ratio" OR PLR OR "C-reactive protein" OR CRP OR "tumor necrosis factor" OR TNF OR interleukin and "Cardiac syndrome X"

Embase

N=157

NLR OR "neutrophil to lymphocyte ratio" OR "platelet to lymphocyte ratio" OR PLR OR "C-reactive protein" OR CRP OR "tumor necrosis factor" OR TNF OR interleukin and "Cardiac syndrome X"

PubMed

N=75

("NLR"[All Fields] OR "neutrophil to lymphocyte ratio"[All Fields] OR "platelet to lymphocyte ratio"[All Fields] OR "PLR"[All Fields] OR "C-reactive protein"[All Fields] OR "CRP"[All Fields] OR "tumor necrosis factor"[All Fields] OR "TNF"[All Fields] OR "interleukin"[All Fields]) AND "cardiac syndrome X"[All Fields]
