# Supplementary material for: Inflammatory biomarkers in cardiac syndrome X: a systematic review and meta-analysis
Source: BMC Cardiovasc Disord. 2024 May 28;24:276. doi: 10.1186/s12872-024-03939-3 (PMC11134643; doi:10.1186/s12872-024-03939-3)
Supplement: Supplementary file 4 — Supplementary Material 4: Supplementary file C shows our data extraction form [file 12872_2024_3939_MOESM4_ESM.docx]

| **Data extraction form** | | | | | | | | |
| --- | --- | --- | --- | --- | --- | --- | --- | --- |
| Number | | | | |  |  |  |  |
| First author | | | | |  |  |  |  |
| published year | | | | |  |  |  |  |
| country | | | | |  |  |  |  |
| Study design | | | | |  |  |  |  |
| Total score | | | | |  |  |  |  |
| sample size | | Total | | |  |  |  |  |
|  |  | CSX | | |  |  |  |  |
|  |  | controls | | |  |  |  |  |
| NLR | CSX | yes | mean± SD | |  |  |  |  |
|  |  |  | Median(Range) | |  |  |  |  |
|  |  |  | Median(IQR) | |  |  |  |  |
|  |  | no | mean± SD | |  |  |  |  |
|  |  |  | Median(Range) | |  |  |  |  |
|  |  |  | Median(IQR) | |  |  |  |  |
| IL-6, | CSX | yes | mean± SD | |  |  |  |  |
|  |  |  | Median(Range) | |  |  |  |  |
|  |  |  | Median(IQR) | |  |  |  |  |
|  |  | No | mean± SD | |  |  |  |  |
|  |  |  | Median(Range) | |  |  |  |  |
|  |  |  | Median(IQR) | |  |  |  |  |
| TNF-*a* | CSX | yes | | mean± SD |  |  |  |  |
|  |  |  |  | Median(Range) |  |  |  |  |
|  |  |  |  | Median(IQR) |  |  |  |  |
|  |  | No | | mean± SD |  |  |  |  |
|  |  |  |  | Median(Range) |  |  |  |  |
|  |  |  |  | Median(IQR) |  |  |  |  |
| PLR | CSX | yes | mean± SD | |  |  |  |  |
|  |  |  | Median(Range) | |  |  |  |  |
|  |  |  | Median(IQR) | |  |  |  |  |
|  |  | no | mean± SD | |  |  |  |  |
|  |  |  | Median(Range) | |  |  |  |  |
|  |  |  | Median(IQR) | |  |  |  |  |
